# Pulsed Electromagnetic Field Stimulation in Osteogenesis and Chondrogenesis: Signaling Pathways and Therapeutic Implications

**DOI:** 10.3390/ijms22020809

**Published:** 2021-01-15

**Authors:** Katia Varani, Fabrizio Vincenzi, Silvia Pasquini, Irene Blo, Simona Salati, Matteo Cadossi, Monica De Mattei

**Affiliations:** 1 Department of Translational Medicine and for Romagna, University of Ferrara, 44121 Ferrara, Italy; vrk@unife.it (K.V.); fabrizio.vincenzi@unife.it (F.V.); psqslv@unife.it (S.P.); 2 Department of Medical Sciences, University of Ferrara, 44121 Ferrara, Italy; irene01.blo@edu.unife.it (I.B.); monica.demattei@unife.it (M.D.M.); 3 IGEA SpA, Clinical Biophysiscs, 41012 Carpi, Italy; m.cadossi@igeamedical.com

**Keywords:** mesenchymal stem cells, pulsed electromagnetic fields, osteogenic differentiation, chondrogenic differentiation, tissue engineering, adenosine receptors

## Abstract

Mesenchymal stem cells (MSCs) are the main cell players in tissue repair and thanks to their self-renewal and multi-lineage differentiation capabilities, they gained significant attention as cell source for tissue engineering (TE) approaches aimed at restoring bone and cartilage defects. Despite significant progress, their therapeutic application remains debated: the TE construct often fails to completely restore the biomechanical properties of the native tissue, leading to poor clinical outcomes in the long term. Pulsed electromagnetic fields (PEMFs) are currently used as a safe and non-invasive treatment to enhance bone healing and to provide joint protection. PEMFs enhance both osteogenic and chondrogenic differentiation of MSCs. Here, we provide extensive review of the signaling pathways modulated by PEMFs during MSCs osteogenic and chondrogenic differentiation. Particular attention has been given to the PEMF-mediated activation of the adenosine signaling and their regulation of the inflammatory response as key player in TE approaches. Overall, the application of PEMFs in tissue repair is foreseen: (1) in vitro: to improve the functional and mechanical properties of the engineered construct; (2) in vivo: (i) to favor graft integration, (ii) to control the local inflammatory response, and (iii) to foster tissue repair from both implanted and resident MSCs cells.

## 1. Introduction

Mesenchymal stem cells (MSCs) are multipotent stromal cells with the ability to self-renew and to differentiate towards osteoblasts, chondrocytes, and adipocytes [1]. Due to their multilineage differentiation potential, MSCs represent an attractive cell source for regenerative medicine approaches. In recent years, tissue engineering (TE) based on the use of MSCs has gained significant attention as an alternative approach to treat bone and cartilage defects. Bone grafts using MSC-based TE approaches have been proposed in a wide array of clinical settings to augment bone repair and regeneration. Likewise, cartilage engineering repair strategies relying on differentiation of MSCs represent an attractive candidate to treat cartilage lesions.

In addition to their multilineage differentiation potential, MSCs have the ability to migrate to injured sites in response to environmental signals and promote tissue regeneration by either directly replacing damaged tissue or interacting with resident cells to promote endogenous repair. The therapeutic effects of MSCs also depend on their ability to control tissue homeostasis: MSCs can modulate the immune response, promote cell survival, and induce angiogenesis through the secretion of growth factors, cytokines, and extracellular vesicles [2]. The ability of MSCs to suppress the immune response is particularly interesting for their clinical application, reducing the chances of rejection by the host immune system after transplantation. Moreover, MSCs are the ideal cellular candidate for TE approaches since they can be easily collected from accessible sources with minimally invasive procedures (e.g., bone marrow, peripheral blood, adipose tissue) and can be rapidly expanded in vitro for clinical use [3]. A recent review from Hassan et al. extensively analyzed the different in vitro expansion protocols for MSCs isolated from different sources: the authors reported that expansion up to 20-fold can be achieved for adipose-derived MSCs (ADMSCs) and bone marrow MSCs (BM-MSCs) without compromising cell viability and differentiation potential, with bioreactor and multi-layered flask being the most effective bioprocessing strategies [4].

Several studies attempted to exploit MSCs in tissue engineering and regenerative medicine approaches; however, in most cases, rates of engraftment were low, engrafted cells were short-lived and failed to fully differentiate into functional terminally differentiated cells. Furthermore, the inflammation frequently characterizing the site of damage further undermines the success of the treatment.

Results from several in vitro and in vivo studies, suggesting that MSCs have the potential to increase bone repair and osteogenesis, have fostered the activation of several registered clinical trials to investigate the role of MSCs in bone defect healing; however, so far, only a few of them have reported their results [5]. The lack of published results causes the effectiveness of these therapeutic approaches to remain controversial.

The application of TE and regenerative medicine approaches to the repair of articular cartilage lesions appear even more complex: although repair strategies that rely on the chondrogenic differentiation of MSCs are attractive, existing methods fail to provide reliable long-term clinical results [6]. Impaired tissue formation, lacking the biological and mechanical properties of the native tissue, together with difficulties in the integration of the engineered construct into the surrounding tissue, have been reported [7]. Consequently, the clinical use of MSCs for cartilage repair, especially for heavy damage such as osteoarthritis, is still debated.

It is therefore clear the need to improve current available TE technologies in order to develop effective tissue substitutes endorsing long-lasting clinical benefits. Along with TE approaches, therapies aimed at either improving the endogenous regenerative capacity of resident MSCs or recruiting to the damaged site repair-competent cells need to be pursued. Treatments capable of improving the performance of MSCs, acting both on their repair/regenerative capacity and their ability to modulate the immune/inflammatory response, will bring significant value to current regenerative medicine and tissue repair approaches.

## 2. Pulsed Electromagnetic Fields

Pulsed electromagnetic fields (PEMFs) are currently applied in the orthopedic field to promote reparative osteogenesis and to provide joint protection [8]. The first report describing the successful application of PEMFs to treat non-union fractures dates back to 1974 [9] and in 1979 the FDA approved PEMFs as a safe and effective treatment for nonunions, congenital pseudoarthrosis, and failed fusions. The scientific bases of PEMFs stimulation for bone healing lie on the identification of the relationship between electrical activity and bone formation in response to applied mechanical load by Fukada and Bassett [10,11].

PEMFs are low frequency magnetic fields, with a specific waveform and amplitude, characterized by a constant variation of the magnetic field amplitude over time. The pulsed magnetic field induces a secondary electric field in the exposed tissue similar to the one naturally generated during the transduction of mechanical energy into electrical energy [12]. In the last 20 years, investigation methodologies adopted from pharmacology have been systematically applied to study the biological effects exerted by PEMFs exposure leading to the development of the physical dynamics, i.e., the science studying the precise combination of physical parameters needed to reach the desired biological effect.

Evidence in the literature shows that the efficacy of a PEMFs stimulation device depends on the physical characteristics of the electromagnetic signal employed. In a survey of the literature, Massari et al. searched for clinical studies evaluating the effectiveness of PEMF treatment. The survey showed that several PEMF devices are available on the market, however only a few PEMF signals are supported by clinical evidence: these signals are either trapezoidal or saw-tooth waves, with magnetic field peak intensity spanning from 1.2 mTesla (mT) to 2 mT and signal repetition frequencies between 15 Hz and 75 Hz [13]. Currently, these devices are used in the clinic to safely promote and accelerate bone fracture healing: PEMFs stimulation has been shown to be effective in promoting the healing of nonunions [14] and delayed unions [15]. Also fresh fractures at risk of nonunion can be successfully treated with PEMFs: Faldini et al., in femur neck fractures, reported 94% healing rate in the PEMFs group compared to 69% in the placebo group [16]. Since the early 2000s, thanks to the results of a large translational research project (Cartilage Repair and Electromagnetic Stimulation: C.R.E.S. study), PEMFs stimulation is also successfully applied to early stage of osteoarthritis, post-traumatic joint pathology, and after surgical treatment of the joints to control inflammation and protect articular cartilage from degeneration [8].

Due to the central role of MSCs in the natural events leading to tissue differentiation and repair and as the cellular components in TE approaches for both bone [17] and cartilage [6], in the last years several efforts have been oriented towards uncovering the electromagnetic fields (EMFs) effects on MSC osteogenic and chondrogenic differentiation as well as the signaling pathways involved. As detailed below, results obtained have shown that PEMFs control inflammatory microenvironment and favor MSC differentiation thus playing a pro-osteogenic and chondrogenic role. In this field, the identification of PEMFs membrane targets, and the specific intra- and extra-cellular pathways involved, allows the rational application of PEMFs to clinical conditions characterized by the disruption of those specific pathways.

## 3. Adenosine Agonist Effect Induced by Pulsed Electromagnetic Fields

Adenosine is a purine endogenous nucleoside with many physiopathological functions involved in cancer, pain, inflammation, and neurodegenerative diseases. Adenosine is primary synthesized from the dephosphorylation of ATP, ADP, and AMP, by the combined action of two hydrolyzing enzymes denominated ectonucleoside triphosphate diphosphohydrolase (CD39) and ecto-5′-nucleotidase (CD73) [18]. Intracellular adenosine levels are maintained low thanks to the conversion to AMP by adenosine kinase. When energy demand rises, for example during inflammation or in hypoxic/ischemic conditions, extracellular adenosine concentration increases [19]. Adenosine functions are mediated by its interaction with four G-protein coupled receptors (GPCRs), namely A_1_, A_2A_, A_2B_, and A_3_ARs. In particular, A_1_ and A_3_ARs are coupled to Gi protein and they inhibit adenylate cyclase (AC) reducing the cAMP levels. Conversely, A_2A_ and A_2B_ARs are coupled to Gs protein and their activation leads to an increase of cAMP [18]. ARs modulation is strongly implicated in the regulation of inflammatory processes suggesting their involvement in different pathologies resulting from inflammation, including many joint diseases [20].

It is well-known that adenosine and its metabolites are important factors in MSC growth and differentiation but they are not always comprised as part of the MSCs secretome [21,22]. Data present in the literature report that adenosine regulates MSCs differentiation stimulating both chondrogenesis and osteogenesis, through A_2A_ and A_2B_ARs activation respectively [23]. The activation of A_2A_ARs by endogenous adenosine controls the cartilage matrix homeostasis in physiological conditions [24]. The loss of A_2A_ARs or CD73, in knockout mice, causes spontaneous osteoarthritis with modified cartilage composition and cartilage thinning [25,26]. Furthermore, it has been reported that A_2A_ARs downregulate osteogenic differentiation and that A_2A_ARs expression is reduced in differentiated osteoblasts, while the contrary occurs during chondrogenesis accompanied by a reduction of CD73. This suggests that changes in A_2A_ARs/CD73 may direct cells toward osteogenic differentiation rather than the chondrogenic one [27].

Similarly to A_2A_ARs, A_3_ARs knockout mice also develop spontaneous osteoarthritis [25,28]. Stimulation of A_3_ARs counteracts inflammation by promoting infiltrating inflammatory cell death and averting chondrocytes apoptosis [29]. In human chondrocytes, A_3_ARs stimulation leads to downregulation of pro-catabolic pathways thus preventing cartilage degeneration [30].

**Figure 1:**
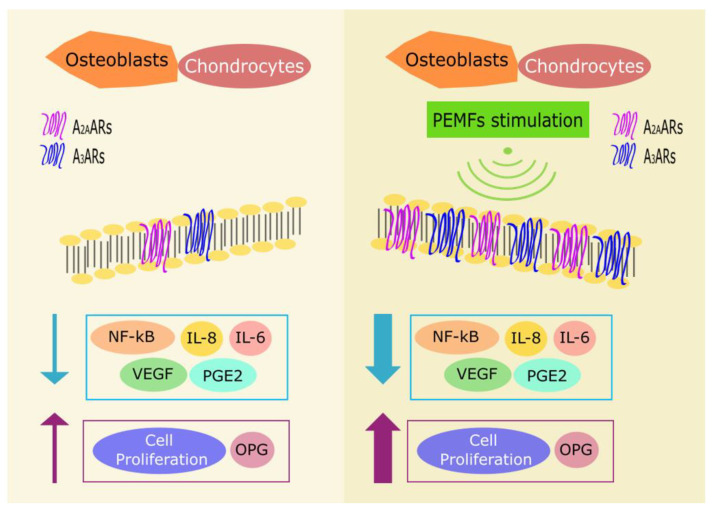
Effect of PEMFs on human osteoblasts and chondrocytes (no PEMFs: left panel, PEMFs exposure: right panel). PEMF stimulation induces an increase of A_2A_ and A_3_ARs expression. The PEMF-induced upregulation enhances the anti-inflammatory downstream receptors signaling by decreasing NF-κB activation and the release of inflammatory mediators, such as IL-6, IL-8, and PGE2. Besides, PEMF-induced modulation of A_2A_ and A_3_ARs results in a reduced VEGF release and increased cell proliferation and OPG production.

The first report describing the effect of PEMFs on ARs dates back to 2002, when Varani et al. reported the upregulation of A_2A_ARs induced by PEMFs exposure in human neutrophils [31]. Later, studies conducted on articular cells—such as chondrocytes and synoviocytes—reported that PEMFs treatment upregulates A_2A_ and A_3_ARs expression (Figure 1) while having no effects on other AR subtypes [32]. The co-treatment with PEMFs and A_2A_ and A_3_AR selective agonists, CGS21680 and Cl-IB-MECA respectively, showed an enhanced effect on the cAMP production in comparison to the agonist treatment alone. This suggests that PEMFs act as modulators able to enhance adenosine agonist activity. This effect was abrogated by using selective A_2A_ and A_3_AR antagonists (SCH 58261 and MRE 3008F20) confirming that the observed effect was due to the activation of A_2A_ and A_3_ARs and not to an alteration of AC functionality [32]. The PEMFs treatment influences also the cellular growth of bovine chondrocytes and fibroblast-like synoviocytes: the co-treatment with CGS21680 and PEMFs significantly increases cell proliferation [32]. Other studies showed that A_2A_ and A_3_ARs stimulation, in the presence of PEMFs, have anti-inflammatory effects decreasing PGE2 release and cyclooxygenase type 2 (COX-2) expression in bovine synovial fibroblasts [33]. As mentioned above, A_2A_ and A_3_ARs have a prominent role in inflammation: treatment with A_2A_ and A_3_AR agonists results in decreased release of pro-inflammatory cytokines such as tumor necrosis factor α (TNF-α), interleukin (IL) 8, PGE2, and IL-6 and increased production of the anti-inflammatory cytokine IL-10 (Figure 1) [34,35]. In vitro studies conducted on T/C-28a2 and hFOB 1.19 cell lines, human chondrocytes and osteoblasts respectively, confirmed previous data obtained in bovine cells revealing that PEMFs exposure leads to augmented expression of A_2A_ and A_3_ARs, as corroborated by RT-PCR, western blotting analysis, and saturation binding experiments [36]. The PEMFs-induced upregulation of A_2A_ and A_3_ARs may reinforce the compensatory mechanism of the body to counteract inflammation. Even in human chondrocytes and osteoblasts, the co-treatment with CGS21680 and PEMFs enhances cell proliferation. Furthermore, both CGS21680 and Cl-IB-MECA showed anti-inflammatory potential decreasing the release of inflammatory cytokines and other mediators implicated in joint inflammation and bone diseases [36]. On the other side, in hFOB 1.19 osteoblasts, PEMF exposure determined an increase of osteoprotegerin (OPG) production (Figure 1). OPG hinders the binding between receptor activator of NF-κB ligand (RANKL) and RANK hampering osteoclasts differentiation and activation [36], thus inhibiting osteolysis.

## 4. Chondrogenic Effects and Pathways Activated by Pulsed Electromagnetic Fields

MSCs are endowed with chondrogenic differentiation potential and represent an attractive cell source for cartilage tissue engineering [1]. Based on their above mentioned pro-chondrogenic and anti-inflammatory effects, PEMFs stimulation could represent an adjuvant strategy to enhance rate and quality of chondrogenic differentiation and to improve the functionality of the repaired tissue ultimately improving the outcome after TE approaches.

PEMFs have been shown to actively promote chondrogenic differentiation of MSCs isolated from different sources. Mayer-Wagner et al. reported for the first time the effect of PEMFs stimulation on the chondrogenic differentiation of human BM-MSCs [37]. In the presence of chondrogenic-inductive growth factors, the exposure to PEMFs induced increased collagen type II (Col2) expression and glycosaminoglycan (GAG) content. Chen et al. reported that PEMFs stimulation enhanced the chondrogenic differentiation of human ADMSCs in both two-dimensional (2D) and three-dimensional (3D) cultures. PEMFs significantly increased the expression of chondrogenic genes (SOX9, collagen type II, and aggrecan) and the deposition of cartilaginous matrix (sulphated GAG) [38]. In human umbilical cord-derived MSCs, Esposito et al. showed that PEMFs enhanced cellular proliferation and chondrogenic differentiation [39]. These results have been confirmed by Kavand et al. in rabbit adipose-derived MSCs cultured in 3D: PEMFs exposure (1.6 mTesla (mT) at 25 or 50 Hz, 8 h/day, over 21 days) increased Col2 expression and extracellular matrix deposition [40]. More recently, Chen et al. showed that PEMFs are effective in promoting the chondrogenesis of superparamagnetic iron oxide nanoparticles (SPIO)-labeled MSCs in a rat model of cartilage defects via activation of the transforming growth factor beta (TGF-β)/SMAD signaling pathway [41].

On the contrary, Wang et al. in rat BM-MSCs reported that PEMFs at 1, 2, and 5 mT inhibit the maintenance of the cartilaginous phenotype and increase cartilage-specific extracellular matrix degradation in the late stage of chondrogenic differentiation [42].

The reported effects of PEMFs on the chondrogenic differentiation of MSCs show a wide variety of responses, from stimulation [37,38] to inhibition of chondrogenesis [42] (Table 1). Significant differences in the PEMFs signal parameters (i.e., waveform, magnetic field peak intensity, frequency, and hours of stimulation) are likely responsible for such discrepancies. In order to overcome this issue, Parate et al. tested the effects of PEMFs exposure on human MSC chondrogenic differentiation by varying magnetic field peak amplitude, exposure duration and dosage. Optimal chondrogenic differentiation was achieved in response to a single 10 min exposure at 2 mT PEMFs: significant upregulation of Sox9, Col2, and aggrecan mRNA expression was reported, which translated into increased chondrogenic ECM deposition after 21 days of culture [43]. The authors also took a significant effort to identify the signaling pathways regulating the response to PEMFs: a time, intensity, and dose-dependent upregulation of both transient receptor potential (TRP) cation channels V4 and C1 (TRPV4 and TRPC1) was detected. The TRP channels have been implicated in cellular mechanotransduction, regulating Ca2+ influx in response to mechanical stimuli. Ca2+ influx is a key event in initiating chondrogenesis, and both TRPV4 and TRPC1 have been involved in chondrogenic differentiation [44] and early chondrocyte expansion [45]. Thus, PEMFs, by recruiting TRP channels, increase intracellular Ca2+ concentration and enhance chondrogenesis.

Increased intracellular Ca2+ concentration has also been involved in the regulation of MSC migration in response to PEMFs [47]. PEMFs exposure (1 mT, 50 Hz, 24 h) was shown to promote MSC migration in an intracellular calcium-dependent manner. PEMFs-induced intracellular Ca2+ increase in turns activates focal adhesion kinase (FAK) signaling, leading to enhanced Rho GTPase activity and increased F-actin network formation promoting cytoskeleton reorganization and cell migration.

Directionalities of the magnetic field have also been shown to impact on MSC chondrogenic differentiation [48]. Celik et al. reported that chondrogenic differentiation was enhanced when MSCs were cultured on randomly oriented scaffolds and exposed to perpendicular PEMFs. This effect was a result of a complex interplay of focal adhesion dynamics, cytoskeleton remodeling, and mitochondrial responses.

PEMFs have also been shown to modulate the MSC secretome, evaluated as conditioned medium (CM) harvested from PEMFs-exposed MSCs [49]. In this study, the authors reported that CM harvested from PEMFs-exposed MSCs (PCM) has increased pro-chondrogenic activity in comparison to medium derived from unexposed cells. In fact, it promoted cartilage formation with superior hyaline phenotype, and showed higher anti-inflammatory potential, protecting cartilage from adverse inflammatory conditions within the articular environment. Moreover, migration of both chondrocytes and MSCs was enhanced by PCM, suggesting its potential to chemotactically attract chondrocytes or MSCs and promote endogenous cartilage regeneration.

The anti-inflammatory effect of PEMFs exposure has been extensively described above. Of particular interest are the results from Ongaro et al. who investigated the effects of PEMFs stimulation during chondrogenic differentiation of bovine synovial MSCs in presence of IL-1β [46]. The authors reported that PEMFs stimulation alone or in presence of chondrogenic-induction medium had a limited effect in promoting chondrocyte differentiation. However, in presence of IL-1β, PEMFs stimulation counteracted the IL-1β-induced inhibition of chondrogenesis, restoring proteoglycan synthesis, and preserving aggrecan and Col2 mRNA expression. These data suggest that PEMFs stimulation sustains and promotes chondrogenic differentiation also under inflammatory conditions.

In agreement with these findings, Veronesi et al. showed that PEMFs stimulation favors osteochondral regeneration in rabbit osteochondral lesions treated with a collagenous scaffold and BM concentrate [50]. In particular, PEMFs exposure significantly improved cartilage cellularity and matrix GAG content, and the macroscopic appearance and percentage of cartilage under the tidemark. More recently, Stefani et al. assessed the effects of PEMFs exposure on the structural and functional quality of tissue-engineered cartilage grafts [51]. The authors reported that PEMFs stimulation promotes the formation of a uniform hyaline-like repaired tissue and reduces the levels of pro-inflammatory cytokines within the joint environment.

## 5. Therapeutic Implications for Cartilage Repair Approaches

The studies discussed above suggest a significant impact of PEMFs stimulation on different aspects of MSC-based therapies for cartilage repair. First of all, PEMFs exposure enhances MSC chondrogenic differentiation through both direct activation of chondrogenic signaling pathways (i.e., TGF-β/SMAD) and indirect paracrine mechanism, mediated by MSC secretome. In this view, PEMFs could be applied as adjuvant therapy to increase cartilage-specific gene expression and chondrogenic differentiation of MSCs to overcome the obstacles of using growth factors in vivo.

Second, PEMFs stimulation can also act as a chemotactic signal for MSCs and chondrocytes thus favoring cell migration to the site of injury to promote tissue repair. Third, PEMFs exert a strong anti-inflammatory effect protecting cartilage tissue from the catabolic activity of pro-inflammatory cytokines. Following cartilage injury or osteoarthritis, the expression of pro-inflammatory cytokines and catabolic factors are upregulated causing sustained inflammation, matrix degradation, and chondrocyte apoptosis [52]. Exposure to PEMFs exerts a direct anti-inflammatory effect through the upregulation of A_2A_ and A_3_ adenosine receptors, thus reducing the release of pro-inflammatory cytokines and increasing the release of anti-inflammatory mediators. In addition, PEMFs exposure increases the anti-inflammatory potential of MSC secretome thus enhancing the therapeutic efficacy of MSCs in mitigating the cartilage damage and restoring the MSC regenerative capacity within the inflamed joint environment.

## 6. Osteogenic Effects and Pathways Activated by Pulsed Electromagnetic Fields

Bone healing is a very complex process including different phases accompanied by the formation of new tissue that restores the injured bone. Following the inflammatory phase, it involves MSCs recruitment, their differentiation into osteoblasts and the production of extracellular bone matrix components. It is now known that all these processes are guided by a plethora of signals that regulate the activities of bone repairing cells, through the modulation of signaling pathways. The main signaling pathways involved have been recently reviewed by Majidinia et al. and include Wnt/β-catenin, Notch, Bone Morphogenetic Protein (BMP)/TGF-β, Phosphoinositide 3-kinases/Akt/mammalian Target Of Rapamycin (PI3K/Akt/mTOR), mitogen-activated protein kinase (MAPK), platelet-derived growth factor (PDGF), insulin-like growth factor (IGF), fibroblast growth factor (FGF), and Ca2+ pathways [53].

Here, we focus on the most relevant data concerning PEMF-induced stimulation of MSC osteogenic differentiation with potential therapeutic implications for the treatment of bone diseases. The EMFs characteristics and effects on osteoblasts and osteoclasts have been recently reviewed by Zhang B et al. [54]. In one of the first studies analyzing the osteogenic effects of PEMFs in human MSCs, it was observed that PEMFs exposure increased the production of osteogenic markers such as alkaline phosphatase (ALP) and osteocalcin (OC) in the presence of Bone Morphogenetic Protein 2 (BMP-2) only, suggesting a synergistic action of PEMFs and BMP-2 [55]. Afterwards, several other authors confirmed that PEMFs stimulate the osteogenic differentiation of MSCs derived from bone marrow or adipose tissue also in the absence of BMP-2 [56,57,58]. The PEMF-induced osteogenic effects have been observed in cells cultured on plastic or other substrates such as nanostructured titanium surfaces (TiO2) [59] and biomaterials [58,60].

While it is commonly recognized that PEMFs exposure induces osteogenic differentiation of MSCs, the signaling pathways underlying such effects are still subject of intensive investigation (Table 2).

The primary cellular target of PEMFs has long been considered the cell membrane. Indeed one of the first discovery concerning PEMFs molecular mechanisms identified changes in Ca2+ fluxes, mainly due to L-type voltage-gated calcium channels (VGCCs) and the consequent activation of the Ca2+/CaM pathway [64]. The increase in intracellular calcium concentration induced by PEMFs has been reported also in hMSCs as an early event during stimulation of osteogenic differentiation [65]. However, to date, the precise mechanism linking PEMFs, calcium and osteogenesis is not completely understood, probably due to the complex mechanisms regulating calcium influx and the still elusive roles of calcium flux and L-VGCCs during osteogenic differentiation [70].

Several studies demonstrated that PEMFs stimulation significantly increases the pro-osteogenic activity of members of the TGF-β gene family, including BMP-2 and -4. Recently, in human bone marrow MSCs (hBM-MSCs), Martini et al. confirmed the combined osteogenic activity of PEMFs and BMP-2 [55,56,66], in the presence of low doses of BMP-2. Furthermore, the authors showed that the PEMFs effects were associated to increased gene expression of several BMP signaling components including BMP-2, BMP-6, BMP type I receptor, and to the activation of SMAD1/5/8, the main player in the canonical BMP signaling pathway [66]. Synergistic effects on osteogenic differentiation of periodontal ligament stem cells (hPDLSCs) have been also observed following combined treatment of PEMFs and BMP-9, another component of the BMP family [63].

Several studies also described the effects of PEMFs stimulation on the Wnt/β-catenin signaling pathway. The involvement of Wnt/β-catenin pathway in the osteogenic differentiation of MSCs induced by PEMFs has been observed in different cellular models [61,69]. Lin et al. (2015) showed that single-pulsed EMF induces osteogenic differentiation in hBM-MSCs by modulating the expression of Wnt signaling pathway components. Specifically, single-pulsed EMF increased the expression of several Wnt ligands, such as Wnt1, Wnt3a, Wnt10b, Fzd9 while downregulating sclerostin, a known Wnt signaling inhibitor [71]. Recently, in the murine mesenchymal stem cell line C3H10T1/2, PEMFs have been shown to induce the gene expression and protein synthesis of components of the Wnt/β-catenin pathway, as well as an increase in intracellular Ca2+ concentration, suggesting a link between Wnt/Ca2+ (non-canonical pathway) and Wnt-β-catenin (canonical pathway) signaling during PEMFs-induced osteogenic differentiation [61].

Other signaling pathways involved in the osteogenic differentiation induced by PEMFs include the MAPK/ERK pathway, also known to be involved in the differentiation of bone cells and bone repair processes. The MAPK signaling pathway includes a class of protein kinases which, through a cascade reaction, activates transcription factors and regulates gene expression and differentiation [53,72]. Several studies have shown the involvement of the MEK/ERK cascade in the osteogenic differentiation induced by PEMFs in human MSCs [57,66]. In hADMSC, Poh et al. reported increased levels of phosphorylated Akt, p70 S6 kinase, S6 ribosomal protein, and ERK1/2 shortly after PEMF exposure, suggesting that a combination of ERK and Akt activation in response to extremely low frequency pulsed electromagnetic fields (ELF-PEMFs) promoted hADMSC growth and survival [57]. On the other side, the activation of p38 MAPK, largely investigated for its role in the modulation of the osteogenic master gene Runx2 [72], has been recently reported during the differentiation of hBM-MSCs induced by PEMFs [66].

Furthermore, activation of PI3K/Akt signaling in hMSCs under PEMFs osteogenic induction has been reported. Zhang et al. described increased levels of phosphorylated Akt, phosphorylated GSK3β, and nuclear β-catenin and indicated the Akt/GSK3β/β-catenin axis involved in osteogenic differentiation induced by PEMFs [54]. Akt involvement has been also reported in hADMSC osteogenic differentiation induced by ELF-PEMFs signals, by using the PathScan Intracellular Signaling Array [57]. In the same cellular model, Akt upregulation has been implicated in the activation of the mTOR pathway identified during PEMFs-induced osteogenic differentiation in both physiological and inflammatory conditions [62].

Notch signaling is another relevant signaling pathway investigated during PEMFs-stimulated osteogenic differentiation [67]. This highly evolutionary conserved pathway is known to be involved in bone healing [73]. In hBM-MSCs, Bagheri et al. showed that PEMFs-enhanced osteogenic differentiation is associated to increased expression of several components of the Notch pathway, such as Notch4, Dll4, Hey1, Hes1, and Hes5. Furthermore, the treatment with Notch inhibitors reduced PEMFs effects, suggesting that activation of Notch pathway is required for PEMF-stimulated osteogenic differentiation [67].

Finally, of particular interest are data reporting the ability of PEMFs to modulate relevant epigenetic regulators such as miRNAs during PEMF-induced osteogenic differentiation of hBM-MSCs. Specifically, De Mattei et al. showed that PEMFs could increase the expression and the extracellular release of miR-26a, miR-29b, miR-218, previously involved in both osteogenesis and angiogenesis [68].

## 7. Therapeutic Implications for Bone Healing

Emerging evidence shows that PEMFs stimulation represents a safe non-invasive approach to favor bone repair and optimize bone tissue engineering. The studies reported above investigated the molecular mechanisms underlying PEMFs effects on MSCs. Globally, the results of these studies show that PEMFs act by modulating signaling pathways with well-established roles in bone repair [53] and open several new perspectives for bone repair therapeutic approaches.

First of all, it appears especially relevant the observation that PEMFs modulate BMP signaling and increase the osteogenic effects of low BMP-2 doses [66]. In fact, as BMP-2 treatment is currently approved for bone therapy, these results support the idea that the combined use of PEMFs and BMP-2 may be useful in orthopedic treatments, with the potential advantage of limiting BMP-2 clinical side effects, probably related to the high BMP-2 doses used, as well as of lowering BMP-2 therapy costs.

Furthermore, several other studies have shown that PEMFs stimulate Wnt signaling pathway, commonly activated during bone healing and currently under investigation as a target for pharmacological intervention. As Wnt pathway is negatively modulated by endogenous molecules such as sclerostin (SOST), molecules targeting SOST, such as SOST antibodies (Scl-Ab) and lithium chloride (LiCl) are currently in clinical trials [71]. On this basis, therapies combining PEMFs with Wnt modulatory molecules can be foreseen [74]. Indeed, combination therapies including treatment with local BMP and antibodies against Wnt signaling inhibitors are currently under investigation.

Similarly, results showing the involvement of Notch signaling in PEMF-induced osteogenic differentiation suggest new bone repair approaches [67]. Although the role of Notch signaling in bone repair has been debated and not completely clarified, some in vivo recent results confirmed a relevant role for this pathway in fracture healing [73]. In agreement with these findings, scaffolds loaded with the Notch ligand Jagged-1 have been shown to favor osteogenesis and promote bone formation in animal models of bone defects [75,76]. Finally, the newly discovered ability of PEMFs to modulate miRNAs involved in osteogenesis deserves further investigation to unravel their potential for translation into therapeutical approaches [68].

## 8. Discussion

**Figure 2:**
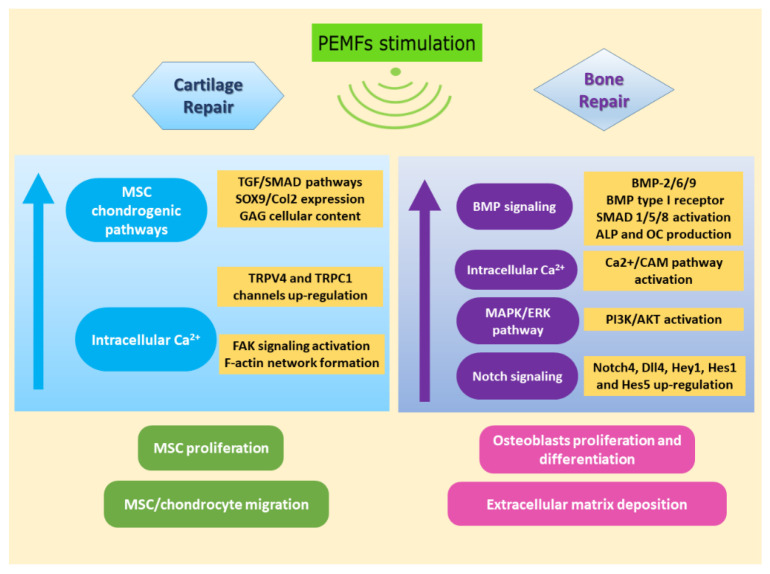
Effects of PEMFs stimulation on MSCs in view of cartilage (left panel) and bone (right panel) repair.

Restoration of critical-size bone defects and repair of articular cartilage lesions pose major challenges to the orthopedic surgeon. Figure 2 shows an overview of PEMFs effects in view of cartilage repair and bone healing.

Bone grafting is a commonly applied surgical procedure for bone repair that employs three types of grafts: autografts, allografts, and synthetic grafts. Tissue engineering represents a promising strategy to overcome the limitations of graft application and the increasing demand for bone grafts. Bone tissue engineering relies on three major players: the cellular component, the scaffold and the osteogenic factors [77]. Collectively, several shreds of evidence support the osteogenic effect of PEMFs on MSCs isolated from different sources, suggesting a synergistic effect with the application of scaffolds and/or osteogenic growth factors [78]. PEMFs can be applied to favor the colonization of the implant with MSCs, promoting their proliferation and osteogenic differentiation [59]. After implantation, PEMFs favor better graft integration and inhibit osteoclast activity, thus protecting the newly formed bone from degradation and enhance the osteogenic regenerative potential of resident MCSs [79]. The recent advances in the knowledge of signaling modulated by PEMFs encourage further investigations on the use of PEMFs alone or in combination with osteogenic molecules and/or biomaterial opening several perspectives for bone repair therapies and optimization of TE approaches.

Cartilage TE for the repair of articular cartilage lesions is an attractive opportunity; however, several limitations still remain: the repaired tissue fails to reproduce the biomechanical properties of the native cartilage thus leading to poor clinical outcomes in the long term [6].

Several factors can undermine the success of the TE procedure, among which local inflammation is one of the most adverse ones. The local microenvironment can significantly impact on in situ degradation, survival and integration of TE constructs, and overall on the success of local repair therapy [6]. Thus, the environment control after the surgical procedure is critical to ensure a successful long-term clinical outcome.

PEMFs have been shown to stimulate cellular proliferation and extracellular matrix deposition favoring the colonization of the engineered construct and the in vitro production of an engineered tissue with better functional and mechanical properties [51]. In vivo, after surgical implantation of the construct, PEMFs stimulation fosters the anabolic activities of MSCs and resident cells and exerts a strong anti-inflammatory effect thus protecting the engineered construct from the detrimental effects of inflammation [51,80].

In various in vitro studies, PEMFs stimulation demonstrated to be effective through the modulation of ARs and the subsequent reduction of inflammatory mediators release [81]. Interestingly, PEMFs through the increase of A_2A_ARs and A_3_ARs potentiate the effect of endogenous adenosine resulting in more physiological effects in comparison to traditional drugs. As a consequence, the PEMF-enhanced anti-inflammatory effect of adenosine would not be accompanied by side effects, receptor desensitization, and downregulation [82]. These observations on adenosinergic system modulation suggest that PEMFs may represent an attractive strategy in the context of ‘soft-pharmacology’ as a non-invasive treatment capable of increasing the effect of an endogenous drug.

PEMFs stimulation has also been reported to promote MSCs migration [47], thus by recruiting endogenous MSCs to the defect site, PEMFs can further improve the chances of success of TE approaches. Lately, the ability of MSCs to affect the surrounding environment through the secretion of a plethora of regulatory molecules has gained significant interest [2]. Through their secretory activity, MSCs are able to exert anti-inflammatory and regenerative activities. PEMFs enhance the secretory activity of MSCs, and in particular, Parate et al. showed that the secretome derived from PEMF-stimulated MSCs could promote cartilage regeneration [49]. These data suggest the possibility to apply PEMFs stimulation to enhance the regenerative potential of MSC-derived secretory products as a cell-free therapeutic for joint injury and osteoarthritis.

Overall, the scientific evidence suggests that tissue repair strategies for the musculoskeletal system should foresee the use of PEMFs both: (1) in vitro: to promote the formation of an engineered construct with improved functional and mechanical properties; (2) in vivo: (i) to promote graft integration; (ii) to locally control the environment after implantation, protecting the engineered construct from the catabolic activity of inflammatory mediators, and (iii) to stimulate tissue repair from implanted and resident cells either through a direct action or through the paracrine effect exerted by MSCs. The discovery of the molecules and the signaling pathways involved in PEMF stimulation will help to define the optimal application of PEMFs towards the development of more effective tissue repair strategies leading to successful and long-lasting clinical outcomes.

## Figures and Tables

**Table 1:** Effects of pulsed electromagnetic fields on the chondrogenic potential of mesenchymal stem cells (MSCs).

Cell Source	PEMF Parameters	PEMF Effects
Rat BM-MSCs	75Hz, 1–5 mT 3 h/day for 4 weeks. Chengdu Miracle Chemical device.	Inhibit the maintenance of the cartilaginous phenotype [42]
Rabbit ADMSCs	75 Hz, 1.8 mT, 8 h/day for 21 days	Increase collagen type II expression and ECM deposition [40]
Bovine MSCs from synovial fluid	Trapezoidal wave, 75 Hz, 1.5 mT, 3–5 weeks. IGEA device.	Counteract the IL-1β-induced inhibition of chondrogenesis [46]
Human ADMSCs	Sinusoidal wave, 1 T, 3 min/day, 3-5-7-10 days.	Increase collagen type II expression and glycosaminoglycan (GAG) content [38]
		Enhance chondrogenic differentiation in 2D and 3D cultures [38]
Human umbilical cord-derived MSCs	Trapezoidal wave, 75 Hz, 1.5 mT, 8 h/day, 21 days. IGEA device.	Enhance cellular proliferation [39]
		Increase chondrogenic differentiation [39]
Human BM-MSCs	Sinusoidal wave, 15 Hz, 5 mT, 45 min every 8 h, for 21 days. TNeue Magnetodyn device.	Increase chondrogenic differentiation [37]
	15 Hz, 1–4 mT, 5–60 min, single and multiple exposures.	Upregulate Sox9, Col2 and aggrecan mRNA expression [43]
		Increase chondrogenic ECM deposition [43]
		Upregulate TRPV4 and TRPC1 [43]
	Sinusoidal wave, 7.5–75 Hz, 1 mT, 24 h. Naval University of Engineering.	Promote MSC migration [47]
		Intracellular Ca2+ increase [47]
		FAK activation [47]
		enhanced Rho GTPase activity [47]
		increased F-actin network formation [47]
	15 Hz, 0–3 mT, 5–30 min, single and multiple exposures.	Perpendicular PEMFs enhance chondrogenic differentiation in MSCs cultured on randomly oriented scaffolds [48]
	15 Hz, 0.5–4 mT, 10 min.	Enhance paracrine function of MSCs for cartilage regeneration [49]

Abbreviations: hours, h; minutes, min; Hertz, Hz; interleukin 1 beta, IL-1β, Focal Adhesion Kinase, FAK.

**Table 2:** Effects of pulsed electromagnetic fields on the osteogenic differentiation of mesenchymal stem cells (MSCs).

Cell Source	PEMF Parameters	PEMF Effects
C3H10T1/2 murine mesenchymal stem cell line	30 Hz, 1 mT, 2 h/day for 20 days. EBI device.	Increase ALP activity, mineralization, Runx2, Osx [61]
		Increase intracellular Ca2+ concentration [61]
		Upregulate Wnt1, phospho-Lrp6, and β-catenin [61]
Human ADMSCs	26 Hz ELF-PEMF 7 min/day for 14 days. Somagen® device.	Increase ALP, mineralization, COL-I and OC gene expression [57] Increase Akt, p70 S6 kinase, S6 ribosomal protein, and ERK1/2 phosphorylation [57]
	12 MHz microwave and 30 mT PEMF, frequency range 50–400 Hz, 8 h/day, 14 days. STRC device.	Increase ALP activity, mineralization, ALP and Runx2 gene expression [58]
	30 days, MED device.	Increase ALP activity, mineralization, ALP and OSP gene expression [62]
		Activate Akt and mTOR pathway [62]
Human periodontal ligament stem cells (hPDLSC)	Rectangular wave, 15 Hz, 1.8 or 2.4 mT, 1 h/day. GHY-III device.	Increase ALP, OPN, mineralization, Runx2 [63]
		Synergistic effect with BMP-9 [63]
Human BM-MSCs	Trapezoidal wave, 75 Hz, 2 mT, 10 min/day for 28 days.	Increase Runx-2, COL-I, FN, OSP, Osx, OC, BMP-2, ALP gene expression; ALP activity; BMP-2, DCN, COL-I protein in cells cultured on nano-TiO2 surfaces [59]
		Increase L-type voltage gated Ca channels (VGCCs) expression [59]
		Increase ALP, COL-I, OPN, DCN proteins [64]
		Increase Ca2+ fluxes by L-type voltage-gated Ca channels (VGCCs) [65]
		Activation of the Ca2+/CaM pathway [65]
		Increase Ca2+ fluxes by L-type voltage-gated Ca channels (VGCCs) [65]
		Activation of the Ca2+/CaM pathway [65]
	Trapezoidal wave, 75 Hz, 1.5 mT, 28 days. IGEA device.	Increase ALP activity, OC production, mineralization, Runx2, Dlx5 [66]
		Activate SMAD1/5/8 and p38 MAPK [66]
		Upregulate BMP-2, BMP-6, BMP type I receptor [66]
		Increase ALP activity, OC level, mineralization, Runx2, Dlx5, Osterix gene expression [67]
		Increase Notch4, Dll4, Hey1, Hes1 and Hes5 expression [67]
		Increase ALP activity, OC production, Runx2 and Dlx5 gene expression [68]
		Increase miR-26a, miR-29b, miR-210 expression or extracellular release [68]
		Increase VEGF expression and release [68]
	Asymmetrical hemi-sine wave, 5-ms pulse every 5 s, 1 T, 3 min/day, days 1–5. Oriental Advance Technology device.	Upregulate Wnt1, Wnt3a, Wnt10b, Fzd9, BMP2 [69]
		Downregulate SOST [69]
		Increase ALP activity, mineralization [69]

Abbreviations: hours, h; minutes, min; Hertz: Hz; ALP, alkaline phosphatase; COL-I, collagen type I; ELF-PEMF, extremely low frequency pulsed electromagnetic fields; FN, fibronectin; OSP, osteopontin; Runx2, runt-related transcription factor 2; Osx, osterix; DCN, decorin; Dlx5, distal-less homeobox 5; VEGF, vascular-endothelial growth factor.

## Data Availability

Data sharing not applicable.

## Abbreviations

MSCs	Mesenchymal Stem Cells
hBM-MSCs	Human Bone Marrow Mesenchymal Stem Cells
hADMSCs	Human Adipose Derived Mesenchymal Stem Cells
hPDLSC	Human periodontal ligament stem cells
PEMFs	Pulsed Electromagnetic Fields
TE	Tissue Engineering
BMP-2	Bone Morphogenetic Protein-2
EMF	Electromagnetic field
BMPs	Bone Morphogenetic Proteins
TGF-β	Transforming Growth Factor beta
MAPKs	Mitogen-Activated Protein Kinases
ALP	Alkaline Phosphatase
OC	Osteocalcin
SOST	Sclerostin
VEGF	Vascular-Endothelial Growth Factor
Ca2+/CaM	Ca2+/Calmodulin
